# Machine Learning–Based Prediction of Breast Cancer in Women: Insights From Feature Selection of Clinical and Lifestyle Data

**DOI:** 10.1155/ijbc/8510185

**Published:** 2026-05-18

**Authors:** Leila Allahqoli, Mohammad Hassan Behzadi, Seyedeh Zahra Aghamohammadi, Sevil Hakimi, Hamid Salehiniya, Arezoo Fallahi, Azam Rahmani, Zahra Shahabinia, Afrooz Mazidimoradi, Zohre Momenimovahed, Mohammadmatin Ghiyasvand

**Affiliations:** ^1^ Faculty of Health Sciences, Cyprus International University, Nicosia, North Cyprus, Cyprus, ciu.edu.tr; ^2^ Department of Statistics, Science and Research Branch, Islamic Azad University, Tehran, Iran, azad.ac.ir; ^3^ Department of Mathematics, Islamshahr Branch, Islamic Azad University, Islamshahr, Iran, azad.ac.ir; ^4^ Faculty of Health Sciences, Ege University, Izmir, Turkey, ege.edu.tr; ^5^ Social Determinants of Health Research Center, Birjand University of Medical Sciences, Birjand, Iran, bums.ac.ir; ^6^ Social Determinants of Health Research Center, Research Institute for Health Development, Kurdistan University of Medical Sciences, Sanandaj, Iran, muk.ac.ir; ^7^ Nursing and Midwifery Care Research Centre, School of Nursing and Midwifery, Tehran University of Medical Sciences, Tehran, Iran, tums.ac.ir; ^8^ Geriatric Health Research Center, Birjand University of Medical Sciences, Birjand, Iran, bums.ac.ir; ^9^ Epidemiology Department, Shiraz University of Medical Sciences, Shiraz, Iran, sums.ac.ir; ^10^ Reproductive Health Department, Qom University of Medical Sciences, Qom, Iran, muq.ac.ir; ^11^ Department of Computer Engineering, Amirkabir University of Technology (AUT), Tehran, Iran, aut.ac.ir

**Keywords:** breast cancer, feature selection, machine learning, predictive modeling

## Abstract

**Objective:**

This study is aimed at developing and evaluating a machine learning–based model for breast cancer classification using integrated clinical, demographic, reproductive, and lifestyle data.

**Methods:**

A retrospective machine learning framework was developed using data from a case–control study conducted in Tehran. The dataset included demographic, clinical, reproductive, lifestyle, and screening‐related variables. Data preprocessing was performed within machine learning pipelines to ensure data quality and prevent data leakage. Duplicate records and noninformative variables were removed. Missing values were imputed using median values for numerical variables and the most frequent value for categorical variables. Categorical features were encoded appropriately, and Min–Max normalization was applied where required. Feature selection was conducted using mutual information (MI) and analysis of variance (ANOVA) within a stratified cross‐validation framework. The data were split into training (80%) and test (20%) sets. Several supervised learning algorithms, including Gaussian Naive Bayes (GNB), K‐nearest neighbors (KNN), decision tree (DT), random forest (RF), support vector machine (SVM), logistic regression (LR), and artificial neural network (ANN), were trained and evaluated. Model performance was assessed using accuracy, precision, recall (sensitivity), F1‐score, and receiver operating characteristic–area under the curve (ROC‐AUC), with stratified 5‐fold cross‐validation and final evaluation on an independent test set.

**Results:**

Significant differences were observed between breast cancer patients and healthy controls across multiple demographic and clinical variables. Patients were generally older and more likely to be widowed, belong to higher socioeconomic classes, and be housewives, whereas higher education levels and employment were more frequent among healthy individuals (*p* < 0.001). Reproductive factors, including age at first marriage and breastfeeding duration, also showed significant differences. Feature selection reduced 414 initial variables to 40 key predictors. The most influential features included genetic factors (BRCA1/2 mutations and family history), reproductive and hormonal characteristics (age at menarche, menopause, and infertility), lifestyle behaviors (dietary patterns and physical activity), anthropometric measures (BMI and weight at age 30), and screening‐related variables (mammography, ultrasound, and biopsy). All models demonstrated strong and stable performance with minimal differences between cross‐validation and test results, indicating good generalization. RF achieved the highest performance (accuracy: 0.9897, precision: 0.9946, recall: 0.9840, F1‐score: 0.9892), followed by SVM and LR, whereas ANN showed the lowest overall performance.

**Conclusion:**

Machine learning models can effectively classify breast cancer using multidimensional patient data. Ensemble methods, particularly RF, demonstrated superior accuracy and robustness, highlighting their ability to capture complex nonlinear relationships. The identified predictors are consistent with established clinical and epidemiological risk factors, supporting the validity of the proposed models. These findings suggest that machine learning approaches hold strong potential for personalized risk assessment and early detection of breast cancer; however, external validation across diverse populations is necessary to confirm generalizability.

## 1. Introduction

Breast cancer is considered one of the most significant public health challenges worldwide and remains the most common cancer among women. According to the GLOBOCAN report in 2022, approximately 2.3 million new cases of breast cancer were diagnosed globally, and nearly 670,000 deaths were reported due to this disease [1]. These statistics highlight the high burden of the disease and its substantial impact on healthcare systems. Furthermore, projections by the International Agency for Research on Cancer (IARC) indicate that, if the current trend continues, the annual number of new breast cancer cases will exceed 3.2 million by 2050, underscoring the urgent need to develop more effective methods for the diagnosis and prediction of this disease [2].

Early detection of breast cancer plays a key role in reducing mortality and improving treatment outcomes; the 5‐year survival rate in the early stages of the disease exceeds 90%, whereas diagnosis at advanced stages is associated with a significant reduction in survival [3]. However, conventional diagnostic methods such as mammography, ultrasonography, MRI, and biopsy, in addition to high costs and the need for specialized equipment, face challenges including false‐positive or false‐negative results, invasiveness of certain procedures, and dependence on expert interpretation [4]. These limitations, particularly in underresourced areas and healthcare centers with limited resources, hinder timely diagnosis.

In many clinical settings, the available data primarily include patients′ clinical features, such as age, family history of cancer, menopausal status, body mass index (BMI) kilograms per square meter, pregnancy and breastfeeding history, hormonal medication use, lifestyle‐related factors, clinical symptoms, and medical records. These data can be collected widely, at low cost, and noninvasively, making them a suitable basis for predictive analyses [5]. Nevertheless, traditional analysis of these data is often unable to uncover complex and nonlinear relationships among variables, which reduces predictive accuracy [6].

In recent years, machine learning, as a branch of artificial intelligence, has fundamentally transformed the analysis of medical data. Machine learning algorithms, with their ability to process multidimensional data and extract hidden patterns, have shown high potential in predicting the risk of breast cancer based on clinical data [7]. Various studies indicate that models such as random forest (RF), support vector machine (SVM), XGBoost, and CatBoost can significantly improve predictive accuracy compared with classical statistical methods [8, 9].

Despite these advances, one of the main challenges in using clinical data is the large number of features and their correlations. Using all available variables without proper feature selection can lead to increased model complexity, overfitting, reduced generalization ability, and decreased interpretability of results [10]. Therefore, the application of effective feature selection methods to identify the most important clinical factors influencing breast cancer prediction is of particular importance [11].

Given the high prevalence of breast cancer, limitations of conventional diagnostic methods, and the wide availability of clinical data, the main issue addressed in this research is how to design an accurate, stable, and interpretable model for predicting breast cancer using only patients′ clinical features and employing machine learning algorithms along with efficient feature selection methods [12]. The aim of this study is to develop a model that can serve as a clinical decision support tool to identify high‐risk individuals early, reduce diagnostic errors, and improve the management of patients with breast cancer.

## 2. Related Work

In recent years, the use of machine learning methods in the prediction and diagnosis of breast cancer has attracted widespread attention as one of the most important application areas at the intersection of data science and medicine. Studies have shown that machine learning–based models can identify hidden patterns associated with the risk of developing breast cancer by utilizing clinical, demographic, and lifestyle data [13, 14]. In a comprehensive systematic review, Hussain et al. reported that most breast cancer prediction models have been developed based on clinical variables and patients′ medical histories, and that algorithms such as logistic regression (LR), SVM, RF, and neural networks have been the most widely used in this field. This study also emphasizes that the quality of data preprocessing and feature selection plays a decisive role in the performance of the models [13].

In a study based on real clinical data in Iran, researchers used a combination of demographic, laboratory, and imaging data to apply various models, including RF and neural networks, for the prediction of breast cancer. The results showed that ensemble models, particularly RF, provide higher accuracy compared with other methods [15]. This finding is consistent with the approach of using multiple algorithms in the present study. Furthermore, in a study by Kalafi et al., machine learning and deep learning models were investigated for predicting the survival of patients with breast cancer using clinical data. The study showed that nonlinear models, such as artificial neural networks (ANNs), are capable of extracting complex relationships among clinical variables and can achieve better performance compared with linear models [16]. In more recent studies, the use of integrated data, including biochemical indicators, has also gained attention. For example, in a 2025 study, machine learning models using clinical and blood‐based biomarker data achieved area under the curve (AUC) values ranging from 0.779 to 0.862 and accuracy between 0.780 and 0.841, highlighting that combining multisource data can improve prediction accuracy [17]. From a methodological perspective, many studies emphasize the importance of data preprocessing steps. In particular, handling missing data, feature normalization, and appropriate encoding of categorical variables have been identified as key factors in improving model performance [13]. This is consistent with the use of standardization and normalization techniques in the present study. In feature selection, numerous studies have shown that statistical filter methods such as analysis of variance (ANOVA) and information‐based measures such as mutual information (MI) are among the most widely used approaches for dimensionality reduction in classification problems. These methods evaluate the statistical relationship between each feature and the target variable, enabling the removal of less important features and improving the efficiency of machine learning models. In particular, in medical datasets, which typically contain a large number of variables and noise, these approaches can enhance model generalizability and reduce overfitting [10]. Additionally, in a multicenter study, researchers used clinical patient data and applied nine different machine learning algorithms, evaluating model performance using metrics such as accuracy, sensitivity, specificity, F1‐score, and AUC. The results showed that ANNs achieved the highest AUC value (0.95), indicating the strong capability of these models in medical classification tasks [18]. Despite significant advances, several challenges remain. Many studies have reported limitations such as data imbalance, small sample sizes, and lack of external validation, which may affect the generalizability of models [13]. In addition, a substantial proportion of research has focused on imaging data, whereas structured clinical data and lifestyle factors—particularly in specific populations—have received less attention. Overall, previous studies indicate that an appropriate combination of data preprocessing, feature selection, and diverse machine learning algorithms can lead to the development of accurate breast cancer prediction models. However, there is still a need for studies focusing on local datasets, lifestyle factors, and demographic characteristics, which has been addressed in the present research.

## 3. Materials and Methods

### 3.1. Data Source and Study Design

This study was conducted in 2025 with the aim of designing and evaluating a machine learning–based model for breast cancer prediction. The present research is a secondary study, and the data used were extracted from a research project conducted by Salehiniya et al., entitled “Development of an appropriate model for estimating the risk of breast cancer in Iranian women (a case–control study).” The data included clinical, demographic, and lifestyle‐related characteristics of Iranian women and were used for two groups: women diagnosed with breast cancer and healthy women. The data collection instrument in the original study was a standardized clinical questionnaire, the validity and reliability of which had been confirmed in that study. In the present research, only the finalized and prepared data were analyzed. The information used included demographic variables; pregnancy history and major life events; family history of women′s diseases; history of medication use and occupational exposures; physical activity level; cigarette, hookah, and alcohol consumption; dietary patterns; and clinical indicators such as height, weight, and weight status during puberty, and at ages 20 and 30 years. All variables were related to the period prior to disease onset. The study population consisted of women with breast cancer who referred to breast cancer diagnosis and treatment centers in Tehran, as well as healthy women as the control group. Sampling in the patient group was performed continuously and proportionally to each center′s share of the total diagnosed cases. The main centers included Payambaran Hospital, the Breast Cancer Research Center of the Academic Center for Education, Culture and Research (ACECR), Shahid Beheshti Cancer Research Center, and Imam Khomeini Hospital. The control group was selected from the general population of Tehran using stratified sampling proportional to the population of urban regions. Inclusion criteria were Iranian nationality, age between 25 and 75 years, residence in Tehran, and willingness to participate in the study. Exclusion criteria included pregnancy, diagnosis of other cancers, receipt of preventive breast cancer treatment, and uncertainty regarding breast cancer status in the control group [19]. Given the case–control design, the proportion of cases and controls in the dataset does not reflect the true population prevalence of breast cancer. Therefore, the developed models are intended for classification purposes rather than direct risk estimation.

### 3.2. Dataset Characteristics

The final dataset included 1932 women, comprising both breast cancer patients and healthy controls. Among the participants, 1000 (51.8%) were classified as healthy controls, whereas 932 (48.2%) were diagnosed with breast cancer. The dataset was relatively balanced, with a nearly equal distribution between the two groups, making it suitable for subsequent machine learning analyses.

### 3.3. Data Preprocessing

Data preprocessing was performed to ensure data quality and support reliable machine learning analysis. This stage included data cleaning, handling missing values, outlier detection, feature encoding, and normalization. The dataset was examined for duplicate records, and duplicates corresponding to the same individual were removed. In addition, variables that did not provide informative value or unnecessarily increased data dimensionality, such as city or province of birth, were excluded.

Missing values were handled within the machine learning pipeline. For consistency across models, median imputation was applied to all numerical variables, whereas categorical variables were imputed using the most frequent value (mode). Outliers were identified and either removed or appropriately handled to preserve data integrity. Binary variables were encoded as 0/1. Nominal categorical variables were transformed using one‐hot encoding. Ordinal variables with a natural order, such as education and socioeconomic status, were encoded according to their inherent ranking. All preprocessing steps, including imputation, encoding, scaling, and feature selection, were performed exclusively within the training pipeline and subsequently applied to the test set to prevent data leakage.

### 3.4. Feature Selection

Feature selection using MI and ANOVA was performed within the training pipeline after data splitting and applied separately in each cross‐validation (CV) fold to prevent data leakage. Selected features were then used for both training and test sets. This was done to ensure that no information from the test set was used during feature ranking.

### 3.5. Modeling

After completing data preprocessing and feature selection, the modeling phase was performed using supervised machine learning algorithms. The objective was to develop predictive models for breast cancer diagnosis based on patients′ clinical and background information. Since the target variable was binary (affected/not affected), the task was formulated as a binary classification problem.

#### 3.5.1. Data Splitting and Normalization

The dataset was divided into two subsets, with 80% of the data allocated for model training and 20% reserved for model testing. Min–Max normalization was applied exclusively to the selected features used in model training, scaling all numeric values to the range [0, 1]. This normalization approach was employed to prevent large numerical differences among features from disproportionately influencing the learning process of the machine learning algorithms.

#### 3.5.2. Machine Learning Algorithms

Several supervised machine learning algorithms were applied to develop predictive models for breast cancer classification. Gaussian Naive Bayes (GNB) was used as a probabilistic classifier based on Bayes′ theorem. The K‐nearest neighbors (KNN) algorithm was implemented as a distance‐based method with nine neighbors. A decision tree (DT) classifier was constructed using the entropy criterion with a maximum depth of 8 and a minimum of 10 samples required for node splitting to control model complexity. RF was employed as an ensemble learning method consisting of 300 DTs with a maximum depth of 10. SVM was implemented using a radial basis function (RBF) kernel to model nonlinear decision boundaries. LR was applied as a linear classification model with L2 regularization. In addition, an ANN based on a multilayer perceptron (MLP) architecture with one hidden layer of 128 neurons and ReLU activation function was used to capture nonlinear relationships in the data, with early stopping and L2 regularization to prevent overfitting.

Data preprocessing and model training were implemented using pipeline structures to avoid data leakage. Missing values were imputed using median values. Feature selection was applied within the pipeline, and scaling using Min–Max normalization was performed for LR, KNN, SVM, and ANN models. Tree‐based models (DT and RF) and GNB did not require feature scaling. Each model was trained on the training set and evaluated on both training and test sets using accuracy, precision, recall, and F1‐score metrics.

#### 3.5.3. Model Performance Evaluation

Performance evaluation was conducted based on the confusion matrix, which includes true positives (TP), true negatives (TN), false positives (FP), and false negatives (FN). Using these components, standard performance metrics were calculated as follows:

Accuracy=TP+TNTP+TN+FP+FN

Precision=TPTP+FP

Recall sensitivity=TPTP+FN

In addition, F1‐score was also calculated as the harmonic mean of precision and recall to provide a balanced measure of model performance, particularly in the presence of class imbalance. Accuracy represents the proportion of correctly classified instances among all observations. Precision indicates the proportion of correctly predicted positive cases among all predicted positives, whereas recall (sensitivity) measures the proportion of actual positive cases that were correctly identified by the model. Sensitivity was emphasized in this study due to the primary objective of accurately identifying patients with breast cancer. Accordingly, the models were compared based on accuracy, precision, and sensitivity to determine the most suitable predictive model. receiver operating characteristic (ROC) analysis and AUC were used to evaluate the discriminative performance of the models. To obtain a robust and unbiased estimate, a stratified 5‐fold CV strategy was applied. In each fold, models were trained on the training subset and evaluated on the validation subset. The independent test set was used only for final evaluation. Final ROC‐AUC performance was reported as mean ± standard deviation across CV folds on the training data, whereas the independent test set was used for final model evaluation.

#### 3.5.4. Overfitting Control

Overfitting was controlled through stratified CV, hyperparameter tuning using grid search, and the use of ensemble learning methods such as RF, which reduces variance by aggregating multiple DTs.

#### 3.5.5. Mathematical Formulation

In this study, the mathematical formulations of two commonly used classifiers, LR and ANN, are presented due to their widespread application in binary classification problems and medical prediction tasks.

##### 3.5.5.1. LR.

LR models the probability of the positive class using the logistic (sigmoid) function:

PY=1X=11+e−β0+∑i=1nβiXi

where *X* = (*X*_1_, *X*_2_, ⋯, *X*_*n*_) represents the input features, *β*_0_ is the intercept, and *β*_*i*_ are the coefficients associated with each feature. The model outputs a probability value, and classification is performed by applying a threshold (typically 0.5).

##### 3.5.5.2. ANN.

ANN used in this study is based on a MLP architecture. Each neuron in the hidden layer computes its output as follows:

$h_{j}=\text{ReLU}\left(\sum_{i=1}^{n}w_{ij}x_{i}+b_{j}\right)\;$ where *x*_*i*_ is the input feature, and *w*_*i* *j*_ is the weight connecting input *i* to neuron *j*, and *b*_*j*_ is the bias term. The ReLU activation function is defined as follows:

ReLU z=max 0,z

The output layer computes the final prediction using the sigmoid activation function as follows:

y∧=11+e−∑jwjhj+b

where $\overset{\wedge }{y}$ represents the predicted probability of the positive class.

### 3.6. Data Analysis Tools

All data preprocessing, feature selection, modeling, and performance evaluation procedures were conducted using the Python programming language. Descriptive statistical analyses were performed using NumPy and Pandas. Quantitative variables were summarized as mean ± standard deviation, whereas categorical variables were reported as frequencies and percentages. Inferential statistical analyses were conducted using SciPy, including independent *t*‐tests for continuous variables and chi‐square tests for categorical variables. A two − sided *p* value < 0.05 was considered statistically significant. Machine learning workflows, including feature selection, data normalization, model training, and performance evaluation, were implemented using the scikit‐learn library. Final model performance was then assessed on an independent test set using accuracy, precision, recall, and F1‐score metrics derived from the confusion matrix. ROC curves and AUC values were computed using predicted probabilities from the models. Visualization of results was performed using Matplotlib and Seaborn libraries.

## 4. Results

The mean age was significantly higher in the cancer group (50.40 ± 9.56 years) compared with the healthy group (42.17 ± 9.49 years). Regarding marital status, the proportion of widowed women was higher in the cancer group (8.6%) than in the healthy group (4.1%). In terms of education, individuals with associate and bachelor′s degrees were more frequent among healthy participants compared with patients (28.4% vs. 21.5%, respectively). Occupational status also showed notable differences, with a higher proportion of housewives in the cancer group (80.0%) compared with the healthy group (72.3%), whereas employment was approximately twice as common in the healthy group. From a socioeconomic perspective, a greater proportion of participants in the cancer group belonged to upper and upper middle socioeconomic classes (14.9% vs. 7.8%). Moreover, the mean age at first marriage was significantly higher in patients (20.15 ± 4.01 years) compared with healthy individuals (17.92 ± 8.09 years) (*p* < 0.001). In contrast, the duration of breastfeeding was significantly longer in the healthy group (23.39 ± 43.46 months) than in the cancer group (24.57 ± 37.23 months) (*p* < 0.001). Overall, never‐married women were more prevalent in the healthy group, whereas higher socioeconomic status and housewife occupation were more common among patients. Detailed comparisons are summarized in Table 1.

**Table 1:** Comparison of baseline and demographic variables between the study groups.

Variable	Healthy group (*n* = 1000)	Cancer group (*n* = 932)	*p* value
Age (years)	42.17 ± 9.49	50.40 ± 9.56	< 0.001 ^∗^
Age at marriage (years)	17.92 ± 8.09	20.15 ± 4.01	< 0.001 ^∗^
Duration of breastfeeding (months)	23.39 ± 43.46	24.57 ± 37.23	< 0.001 ^∗^
Marital status	729 (72.9)	744 (79.8)	< 0.001 ^∗∗^
Married	133 (13.3)	61 (6.5)	
Never married	34 (3.4)	47 (5.0)	
Divorced			
Widowed	41 (4.1)	80 (8.6)	
Education	48 (4.8)	55 (5.9)	< 0.001 ^∗∗^
Illiterate	108 (10.8)	163 (17.5)	
Primary	158 (15.8)	148 (15.9)	
Middle school	342 (34.2)	316 (33.9)	
High school	284 (28.4)	200 (21.5)	
Associate/bachelor	60 (6.0)	50 (5.4)	
Master and above			
Occupation	723 (72.3)	723 (80.0)	< 0.001 ^∗∗^
Housewife	159 (15.9)	96 (10.3)	
Government employee	108 (10.8)	48 (5.2)	
Private sector	10 (1.0)	42 (4.5)	
Retired			
Insurance	107 (10.7)	32 (3.4)	< 0.001 ^∗∗^
No	839 (89.3)	900 (96.6)	
Yes			
Socioeconomic class	78 (7.8)	139 (14.9)	< 0.001 ^∗∗^
	563 (56.3)	636 (68.2)	
High and upper middle	359 (35.9)	157 (16.8)	
Middle			
Lower middle and low			

*Note:* Continuous variables are presented as mean ± standard deviation (SD), whereas categorical variables are reported as number and percentage (*n*%).

^∗^Independent *t*‐test.

^∗∗^Chi‐square test.

### 4.1. Data Preparation and Feature Selection Outcomes

The initial dataset contained 1932 records. During preprocessing, duplicate records and noninformative variables were identified and removed. Specifically, variables related to place of birth and other nonanalytical attributes were excluded to maintain focus on clinically and epidemiologically relevant features. Missing data were systematically examined, and variables with more than 30% missing values were removed. For the remaining variables, missing values were imputed using the mean for normally distributed numeric variables, the median for nonnormally distributed numeric variables, and the mode for categorical variables. This approach preserved sample size while minimizing potential bias. Outliers were identified and implausible values inconsistent with clinical context were corrected or excluded. All binary and multilevel categorical variables were encoded using appropriate encoding schemes, including binary encoding for dichotomous variables and one‐hot or label encoding for multiclass variables. After initial preprocessing, 414 features were available. Feature selection was then applied within a CV framework, resulting in 40 features used for model training. Feature selection was performed using MI and ANOVA filter‐based methods to rank variables based on their association with the target outcome.

Table 2 presents the 30 highest scoring features identified through the feature selection process. The updated results indicate that genetic and familial factors, particularly history of BRCA1/BRCA2 gene mutations and family history of breast cancer, were among the most influential variables in distinguishing breast cancer patients from healthy individuals. In addition, reproductive and hormonal characteristics, including age at menarche, age at first marriage, age at first live birth, menstrual cycle characteristics, natural and surgically induced menopause, and infertility history, showed strong predictive importance.

**Table 2:** This table lists the 30 features with the highest scores.

Number	Score	Standardized description
1	322	History of BRCA1/BRCA2 gene mutation
2	322	Frequency of coffee consumption
3	319	Family history of breast cancer in first‐degree relatives
4	316	Weekly frequency of egg consumption
5	311	Body weight at age 30
6	309	History of breast biopsy prior to symptom onset
7	309	Current age
8	309	Residential area (proxy for socioeconomic and geographic status)
9	304	Average length of menstrual cycle (days)
10	304	History of mammography prior to symptom onset
11	303	Age at menarche
12	293	Native language: Persian
13	292	History of breast ultrasound prior to symptom onset
14	280	Menopause due to bilateral oophorectomy
15	277	Family history of breast cancer in second‐ or third‐degree relatives
16	274	History of migration (relocation to another city or country)
17	272	Natural menopause
18	271	History of infertility
19	269	Native language: Turkish
20	269	Predominant type of dietary fat used for cooking
21	267	Body mass index (BMI)
22	266	Age at first marriage
23	264	Frequency of vegetable consumption
24	260	Age at first live birth
25	258	Level of weekly physical activity
26	257	Frequency of fruit consumption
27	256	Total number of pregnancies
28	254	History of breastfeeding
29	253	Frequency of yogurt consumption
30	250	Family history of nonbreast, nonovarian cancers in first‐degree relatives

Lifestyle‐related factors also contributed substantially to model discrimination. These included frequency of coffee, egg, vegetable, fruit, and yogurt consumption, predominant dietary fat type, and overall physical activity level. Anthropometric and demographic variables such as BMI kilograms per square meter, body weight at age 30, current age, and residential area were also ranked among the top features.

Moreover, breast cancer screening‐related variables, including history of mammography, breast ultrasound, and breast biopsy prior to symptom onset, were identified as important predictors. Additional variables such as migration history, native language (Persian and Turkish), and family history of nonbreast and nonovarian cancers were also included among the top‐ranked features.

Overall, the updated feature set highlights the multifactorial nature of breast cancer risk, involving genetic predisposition, reproductive and hormonal history, lifestyle and dietary habits, screening history, and socioeconomic and demographic factors.

### 4.2. Machine Learning Model Performance

The results presented in Table 3 indicate that all evaluated machine learning models demonstrate generally strong and stable performance across both CV and independent test sets, with relatively small differences between the two evaluation strategies in most cases, suggesting an overall good generalization ability and limited overfitting.

**Table 3:** Detailed quantitative comparison of all performance metrics for the evaluated model.

Model	CV accuracy	CV precision	CV recall	CV F1‐score	Test accuracy	Test precision	Test recall	Test F1‐score
LR	0.9359	0.9378	0.9289	0.9332	0.9483	0.9613	0.9305	0.9457
DT	0.9288	0.9065	0.9503	0.9279	0.9354	0.9091	0.9626	0.9351
RF	0.9786	0.9747	0.9812	0.9779	0.9897	0.9946	0.9840	0.9892
KNN	0.9275	0.9434	0.9047	0.9232	0.9354	0.9602	0.9037	0.9311
SVM	0.9476	0.9538	0.9369	0.9452	0.9587	0.9777	0.9358	0.9563
GNB	0.9184	0.9148	0.9181	0.9159	0.9457	0.9611	0.9251	0.9428
ANN	0.9165	0.9203	0.9060	0.9128	0.9199	0.9239	0.9091	0.9164

Abbreviations: ANN, artificial neural network; DT, decision tree; GNB, Gaussian Naive Bayes; KNN, k‐nearest neighbors; LR, logistic regression; RF, random forest; SVM, support vector machine.

Among all models, RF consistently achieves the best performance across all metrics. It shows a very high and stable accuracy, increasing slightly from 0.9786 in CV to 0.9897 in the test set, whereas F1‐score also remains consistently high (from 0.9779 to 0.9892). This minimal variation indicates strong robustness and excellent generalization capability. LR also exhibits stable behavior, with a modest improvement in test accuracy (0.9483) compared with CV (0.9359). Precision increases more noticeably from 0.9378 to 0.9613, whereas recall remains relatively stable around 0.93, suggesting a slightly improved ability to reduce false positives in the test phase. SVM demonstrates a similar pattern, with improved test accuracy (0.9587 vs. 0.9476 in CV) and higher precision (0.9777), whereas recall remains relatively stable (~0.936). This indicates a tendency toward more conservative classification, favoring precision over sensitivity. KNN shows comparatively lower recall in the test set (0.9037), despite maintaining high precision (0.9602). This imbalance suggests a more conservative decision boundary, leading to missed positive cases, although overall accuracy remains moderate and stable across CV and test. DT presents an interesting pattern, where recall increases in the test set (0.9626 compared with 0.9503 in CV), whereas precision slightly decreases. This reflects a shift toward higher sensitivity at the cost of reduced precision, indicating a tendency to classify more instances as positive. NB shows improved performance in the test set compared with CV, with accuracy increasing from 0.9184 to 0.9457 and consistent improvements in precision. This suggests that the probabilistic assumptions of the model align reasonably well with the underlying distribution of the test data. In contrast, the ANN demonstrates the weakest overall performance among the evaluated models, with minimal variation between CV and test results (accuracy around 0.916–0.920 and F1‐score around 0.913–0.916), indicating limited advantage over simpler models in this specific dataset.

Overall, the differences between CV and test results across most models remain small (generally within 0.01–0.02), indicating good generalization and minimal overfitting. However, ensemble‐based methods, particularly RF, consistently outperform other approaches in terms of both stability and predictive performance.

**Figure 1:**
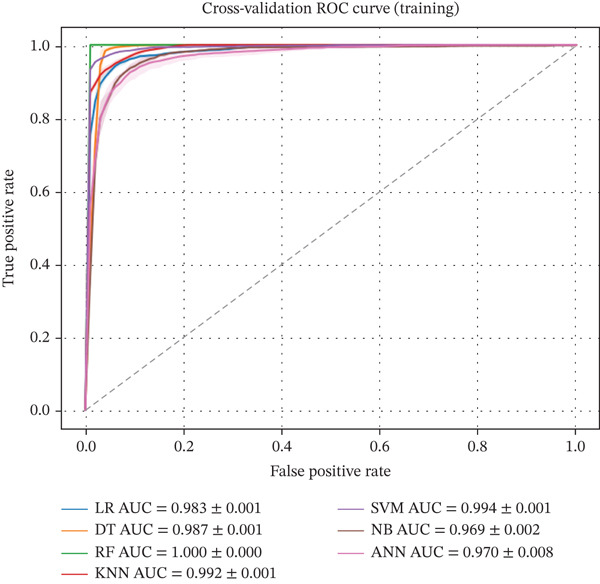
Cross‐validated ROC curves for training sets across all machine learning models, showing mean ROC performance with AUC (mean ± SD) obtained from stratified 5‐fold cross‐validation.

**Figure 2:**
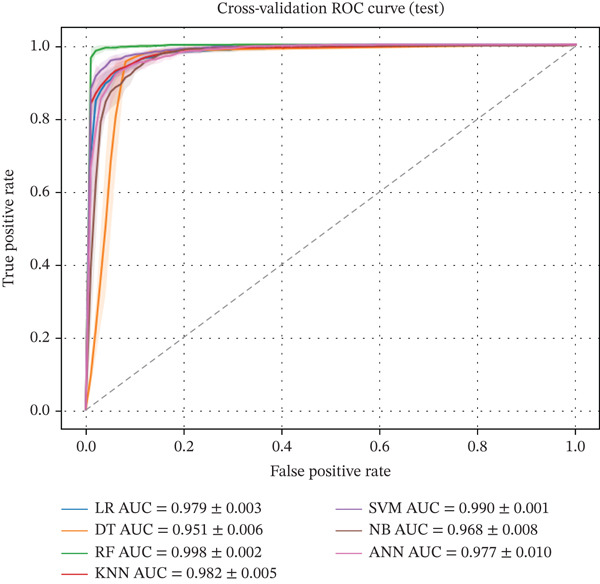
Cross‐validated ROC curves testing sets across all machine learning models, showing mean ROC performance with AUC (mean ± SD) obtained from stratified 5‐fold cross‐validation.

Figures 1 and 2 illustrate the CV ROC curves of the applied machine learning models for the test and training partitions. The curves represent the mean ROC across stratified k‐fold CV folds. The corresponding AUC values are reported as mean ± standard deviation, reflecting the stability and discriminative ability of each model across different data splits.

## 5. Discussion

In the present study, several supervised machine learning algorithms were evaluated for breast cancer classification using a combination of demographic, clinical, and lifestyle‐related features. Overall, all models demonstrated strong predictive performance with relatively small differences between CV and test results, indicating good generalization ability and limited overfitting. Among all evaluated models, RF consistently achieved the best performance across all metrics, with a test accuracy of 0.9897, precision of 0.9946, recall of 0.9840, and F1‐score of 0.9892. The strong performance of RF is consistent with its theoretical advantages in handling high‐dimensional and heterogeneous clinical datasets through ensemble averaging, which reduces variance and improves robustness [20]. Similar conclusions have been reported by Breiman, who originally introduced RF and demonstrated its superior performance compared with single DTs in complex datasets [21]. Furthermore, ensemble learning methods have been widely shown to outperform individual classifiers in medical prediction tasks due to their ability to reduce overfitting and improve stability [22].

SVM and LR also demonstrated strong and competitive performance. SVM achieved a test accuracy of 0.9587 and F1‐score of 0.9563, whereas LR reached 0.9483 accuracy and 0.9457 F1‐score. The strong performance of SVM in this study is consistent with its well‐established capability in handling high‐dimensional biomedical data and nonlinear decision boundaries [23]. Previous studies have also reported that SVM performs competitively in breast cancer classification tasks, often achieving accuracy above 90% when properly tuned [24]. LR, despite its simplicity, remains a strong baseline model in clinical prediction due to its interpretability and robustness, particularly when relationships between variables are approximately linear [25]. In contrast, DT and KNN showed slightly lower and more variable performance. DT achieved a test accuracy of 0.9354 with higher recall (0.9626) but lower precision (0.9091), indicating a tendency toward higher sensitivity at the cost of increased false positives. This behavior is consistent with previous findings showing that single DTs are prone to overfitting and instability when data complexity increases [26]. KNN showed a similar pattern, with relatively high precision (0.9602) but reduced recall (0.9037), suggesting limited sensitivity in detecting all positive cases. This limitation is consistent with the known sensitivity of KNN to feature scaling and class distribution imbalance, as well as its reliance on local distance structures, which can be unstable in high‐dimensional medical datasets [27]. NB achieved moderate performance (accuracy = 0.9457, F1 − score = 0.9428), reflecting reasonable classification ability despite its strong independence assumptions. Although NB often performs surprisingly well in medical datasets, its assumption of conditional independence between features may limit performance when strong feature correlations exist [28].

ANN showed the lowest performance among all models (accuracy = 0.9199, F1 − score = 0.9164). This result suggests that the applied network architecture did not provide a significant advantage over simpler models in this structured dataset. This finding is consistent with prior literature indicating that ANN performance is highly dependent on dataset size, feature representation, and hyperparameter optimization, and does not always outperform classical machine learning models in tabular clinical data [29].

Overall, the small gap between CV and test performance across all models (generally within 0.01–0.02) indicates strong model generalizability and suggests that overfitting was effectively controlled through preprocessing, feature selection, and CV strategies. These results align with previous systematic reviews showing that multiple machine learning algorithms—including LR, SVM, RF, and KNN—typically achieve high accuracy (often above 90%) in breast cancer classification tasks when trained on well‐structured clinical datasets [30]. From a clinical perspective, the most important outcome of this study is the strong performance of ensemble‐based models, particularly RF, which demonstrated both high accuracy and a balanced sensitivity‐specificity trade‐off. This suggests that integrating heterogeneous patient data (demographic, reproductive, genetic, and lifestyle factors) can significantly improve predictive performance when combined with robust ensemble learning techniques. These findings support the growing evidence that machine learning models can serve as effective decision‐support tools in breast cancer risk assessment and early diagnosis [31].

## 6. Limitations and Future Research

Despite the promising performance of the developed machine learning models, several limitations should be acknowledged. First, this study was based on a secondary case–control dataset collected from a specific population of Iranian women in Tehran, which may limit the generalizability of the findings to other populations with different demographic, genetic, and lifestyle characteristics. Second, the retrospective nature of the dataset and reliance on self‐reported variables may introduce recall bias and measurement inaccuracies, particularly for lifestyle and reproductive factors. In addition, although extensive preprocessing and feature selection techniques were applied, the possibility of residual confounding and unmeasured variables cannot be completely excluded. Third, the study was limited to structured clinical, demographic, and lifestyle variables and did not include imaging data (e.g., mammography) or molecular/genomic information, which are known to play an important role in breast cancer detection and risk assessment. Finally, although CV and an independent test set were used to evaluate model performance, external validation on independent cohorts was not performed, which is necessary to confirm the robustness and clinical applicability of the proposed models.

## 7. Conclusion

The findings of this study demonstrate that machine learning approaches can achieve high accuracy and stability in breast cancer classification when applied to well‐structured clinical, demographic, and lifestyle datasets. Among the evaluated models, RF consistently outperformed other algorithms across all performance metrics, exhibiting both high predictive accuracy and strong generalization, as evidenced by the minimal gap between CV and test results. Linear models such as LR and margin‐based methods like SVM also showed competitive performance, indicating that both simple and complex algorithms can effectively capture the underlying patterns in the data. In contrast, models such as KNN, DT, and ANN displayed relatively lower or more variable performance, suggesting sensitivity to data structure, feature scaling, and model configuration. Overall, the consistency between training and test results across models indicates that overfitting was effectively controlled through appropriate preprocessing, feature selection, and validation strategies.

Beyond predictive performance, this study highlights the multifactorial nature of breast cancer risk, with key predictors spanning genetic susceptibility, reproductive and hormonal factors, lifestyle behaviors, and screening history. The identification of variables such as BRCA mutations, family history, body weight across life stages, physical activity, and screening practices underscores the clinical relevance and external validity of the models. These findings suggest that integrating heterogeneous patient data within machine learning frameworks can provide a comprehensive and clinically meaningful approach to risk stratification. Such models have strong potential for application in personalized screening programs and decision‐support systems, ultimately contributing to earlier detection and improved patient outcomes. Future research should focus on external validation across diverse populations and the integration of additional data sources, such as imaging and genomic information, to further enhance predictive performance and clinical applicability.

## Author Contributions

L.A. and M.G. contributed to the conceptualization, study design, and data analysis of the secondary dataset. M.H.B. acted as the study supervisor and provided critical guidance throughout the research process. S.Z.A., S.H., A.F., A.R., Z.S., A.M., and Z.M. contributed to data verification, interpretation, and critical discussion. The initial manuscript draft was prepared by L.A., M.G., and S.Z.A., with subsequent revisions and critical review provided by M.H.B., S.H., and H.S.

## Funding

No funding was received for this manuscript.

## Disclosure

All authors read, critically reviewed, and approved the final version of the manuscript.

## Ethics Statement

The authors have nothing to report.

## Consent

The authors have nothing to report.

## Conflicts of Interest

The authors declare no conflicts of interest.

## Data Availability

The data supporting the findings of this study are available from the corresponding authors upon reasonable request.
